# Management of Pediatric Genitourinary Anomalies: Navigating the Borderline Between Pediatric Surgery and Pediatric Urology

**DOI:** 10.7759/cureus.90702

**Published:** 2025-08-21

**Authors:** Quadri A Sanni, Remi Solagbade, Aminat Giwa, Waliyu I Aro, James Tsejime, Ejike Anyaegbu, Moazam Mehmood

**Affiliations:** 1 Urology, South Warwickshire University NHS Foundation Trust, Warwick, GBR; 2 Urology, University of Ilorin Teaching Hospital, Ilorin, NGA; 3 General Medicine, Central and North West London NHS Foundation Trust, London, GBR; 4 General Medicine, NES, London, GBR; 5 General Surgery, South Warwickshire University NHS Foundation Trust, Warwick, GBR; 6 Emergency Medicine, South Warwickshire University NHS Foundation Trust, Warwick, GBR

**Keywords:** cryptochordism, hypospadias repair, pediatric surgery, pediatric urology, posterior urethral valves

## Abstract

Pediatric surgery and pediatric urology are two surgical specialties that are interwoven yet have different career paths. This review aims to elaborate on the interactions and various models of practice between pediatric surgery and pediatric urology across different continents. A literature search was done using different databases, including PubMed, Medline, Scopus, and Google Scholar. There is significant overlap between pediatric surgery and pediatric urology in the management of pediatric congenital and acquired genitourinary conditions. On the subcontinent in particular, training pathways and availability of these specialties depend largely on subregional protocols. Generally, there are distinct training, clinic, and referral pathways in high-income countries (HICs) as opposed to low- and middle-income countries (LMICs), where there is a severe shortage of specialists and less distinct training guidelines. The intersection between pediatric urology and pediatric surgery represents a dynamic and evolving frontier in pediatric surgical care. While the two specialties are distinct in their training and focus, they frequently converge in managing a wide range of congenital and acquired genitourinary conditions in children.

## Introduction and background

Pediatric urology and pediatric surgery are surgical specialties that are closely related and focus on the care of infants and children. Although they share overlapping domains, especially in the management of congenital and acquired genitourinary conditions, they significantly differ in scope, training, and depth of focus [1,2]. Pediatric surgery involves the surgical management of various conditions in children, from neonatal emergencies like intestinal atresia and congenital diaphragmatic hernia (CDH) to gastrointestinal, hepatobiliary, thoracic, oncologic, and genitourinary anomalies [3]. Pediatric surgeons are skilled in handling diverse cases, often serving as the first point of evaluation and surgical intervention for urological conditions, especially in regions with limited access to pediatric urologists. Pediatric urology, a subspecialty of either adult urology or pediatric surgery, specializes in diagnosing and surgically treating congenital and acquired genitourinary disorders in children [2,4]. Key areas of focus include hypospadias, vesicoureteral reflux, posterior urethral valves, neurogenic bladder, and complex renal or bladder reconstruction [5]. Pediatric urologists receive specialized fellowship training in advanced techniques such as laparoscopy, endourology, and urodynamic assessment. Their expertise is essential for managing intricate urological conditions that demand precise surgical skills and long-term follow-up [6,7].

The overlap between pediatric surgery and pediatric urology is evident in conditions like hypospadias repair, urinary tract obstruction management (e.g., posterior urethral valves), and orchidopexy for undescended testes [8]. The choice of specialist depends on factors such as healthcare system structure, institutional protocols, specialist availability, case complexity, and regional training models. This variability is particularly pronounced between low- and middle-income countries (LMICs) and high-income countries (HICs). While collaboration between the two specialties exists, unclear boundaries can result in fragmented care, delayed referrals, and inconsistent surgical outcomes. This narrative review examines the overlap between pediatric surgery and pediatric urology in terms of clinical practice, training, and the care system; the factors shaping their practice boundaries; and how improved collaboration and role clarity can enhance patient care for urological conditions.

## Review

Historical context

The field of pediatric surgical care developed in the mid-20th century, driven by progress in anesthesia and neonatal medicine [9,10]. By the 1950s and 1970s, it had become an established specialty in many HICs [1,11]. In the UK, for example, the appointment of surgeon Dennis Browne at the Great Ormond Street Hospital in 1928 marked the formal beginning of pediatric surgery in England [12]. Similarly, in North America, Dr. William Ladd is regarded as the pioneer of pediatric surgery in the 1920s [13,14].

Pediatric urology emerged later as a subspecialty, initially developing within adult urology [15]. Its recognition grew due to the increasing complexity of congenital urological conditions and the demand for specialized expertise in reconstructive and functional urogenital surgery for children [2]. Formal training programs in pediatric urology only began in the 1980s, primarily in North America and Europe [2,16]. Historically, the distinction between pediatric surgery and pediatric urology has been flexible [17]. In many areas, general pediatric surgeons handled urological cases due to a shortage of subspecialists. However, as pediatric urology became more defined, especially in tertiary care centers, clearer boundaries developed, though significant overlap remains [17].

Training Pathways and Certification

Training and certification structures vary across regions. In the US and Canada, pediatric urology is a recognized subspecialty of adult urology, requiring completion of a urology residency followed by a one- to two-year fellowship. Certification is granted by the American Board of Urology (ABU) in the US and the Royal College of Physicians and Surgeons in Canada [14,18]. In the UK, pediatric surgery training includes foundational urology during residency, with further subspecialization often requiring additional fellowships [19]. Some pediatric urologists also transition from adult urology. The British Association of Pediatric Urologists (BAPU) oversees professional standards [1,20].

Across Europe, training models differ by country. The European Society for Pediatric Urology (ESPU) promotes a multidisciplinary approach, welcoming practitioners from both pediatric surgery and urology backgrounds. It also works to standardize training through European guidelines [20,21]. In LMICs, access to pediatric urologists is limited, so general pediatric surgeons often manage urological cases. Subspecialization pathways are usually informal or supported by external programs. Due to workforce shortages, pediatric surgery remains the primary specialty for most pediatric genitourinary procedures [22].

Global Practice Model

There are models of practice between pediatric surgeons and pediatric urologists. In the urology-led model, which is found in North America and some parts of Europe, pediatric urologists lead the care for most genitourinary conditions, and there are clear and distinct pathways between pediatricians and surgeons [2,23]. There is the surgery-led model, which is common in the United Kingdom, sub-Saharan Africa, and South Asia. In this model, general pediatric surgeons manage both general and urological surgical cases, and subspecialist referral is less common or not available [11,24]. Finally, there is the hybrid/collaborative model, where there are joint pediatric surgery and urologic clinics, and it is common in big teaching hospitals [24]. The variability in training and role delineation creates inconsistencies in care and training exposure, and operative outcomes [25].

Areas of clinical and surgical overlap

Pediatric surgeons and pediatric urologists have areas of shared responsibility in managing genitourinary conditions [21]. While some cases fall within one specialty domain, many others lie in the ‘grey zone’ where either specialist may be involved, depending on training, experience, policies of the practicing institution, and regional availability. Three genitourinary conditions, such as hypospadias, cryptorchidism, and posterior urethral valves (PUVs), exemplify areas of collaborative care and challenges in defining surgical boundaries.

Hypospadias

Hypospadias is a congenital condition where the urethral meatus is abnormally positioned along the ventral aspect of the penis. The severity ranges from distal (glanular) to proximal (penoscrotal or perineal) [26,27]. In this clinical condition, distal hypospadias may be repaired by both pediatric surgeons and urologists, while proximal and complex hypospadias often require advanced reconstructive skills, and it is more commonly managed by pediatric urologists [27-29]. In some settings, pediatric surgeons handle most hypospadias cases due to a lack of subspecialists [22,30,31]. Some studies suggest that there is no significant difference in outcomes following hypospadias repair [32]. There are, however, areas of controversy, including optimal age of repair, use of one-stage or two-stage procedures, and exposure during residency training [33,34].

Undescended Testes (Cryptorchidism)

Undescended testes are among the most common congenital anomalies in male infants, which are typically managed with orchidopexy [35]. Both pediatric surgeons and pediatric urologists routinely manage palpable testes. Non-palpable or intra-abdominal testes may require laparoscopy and more specialized evaluation, often by urologists in tertiary centers [36]. There are, however, overlapping considerations. The timing of surgery, as guidelines recommend, is six to 12 months of age. Controversies surround contralateral exploration, testicular atrophy risk, and long-term fertility concerns [37-39]. General surgeons in low-resource settings often perform this surgery due to workforce limitations [40]. Orchidopexy is considered a core competency for both specialties during training [41].

Posterior Urethral Valves

Posterior urethral valves are a common cause of lower urinary tract obstruction in male neonates. Prompt diagnosis and decompression are critical to prevent renal damage. Initial decompression with catheterization or nephrostomy may be managed by either specialty [42-44]. Definitive management via valve ablation or endoscopy is more often performed by pediatric urologists, particularly in centers with access to neonatal endoscopy [45]. In many LMICs, pediatric surgeons handle both initial and definitive care [46]. Further management, such as bladder dysfunction, reflux, and renal insufficiency, typically falls under urology, highlighting the need for long-term collaboration. ​​Complex cases require access to pediatric cystoscopy equipment, training in endoscopic techniques, and multidisciplinary nephro-urology follow-up [45]. Other areas of joint management involving pediatric urologists and pediatric surgeons include vesicoureteric reflux, antenatal hydronephrosis, and neurogenic bladder. 

Multidisciplinary approaches and collaborative models

As pediatric urology and pediatric surgery increasingly intersect in clinical practice, multidisciplinary approaches have become essential in managing shared surgical domains [2]. These models aim to enhance patient outcomes, reduce fragmented care, and ensure timely referral and operative planning. Collaboration also mitigates the impact of training variability and workforce shortages.

Rationale for Collaboration

Complexity of Care, in which many urological conditions in children (e.g., PUVs, neurogenic bladder) require both surgical intervention and long-term medical follow-up [45]. In addition, there is shared responsibility in conditions like hypospadias and cryptorchidism, which are often managed by both pediatric surgeons and urologists, depending on institutional capacity, continuity, and coordination to ensure proper follow-up and promote efficiency in surgical planning.

Models of Collaborative Practice

Joint pediatric urology-surgery clinics: This is where both specialties share outpatient clinics and where both specialists evaluate patients. This provides the benefit of unified treatment plans and streamlined patient experience, and avoids contradictory opinions from separate assessments. Such clinics can be effective for treating cases with complex genital anomalies and antenatal genitourinary abnormalities [47].

Multidisciplinary teams (MDTs): These teams would include pediatric surgeons, pediatric urologists, pediatricians, pediatric nephrologists, radiologists, and pediatric nurse specialists. The MDT meetings facilitate shared decision-making, joint postoperative surveillance plans, and consensus on operative indication and timing [45].

Surgical co-management in the operating theater: In complex cases, co-surgery by both pediatric urologists and surgeons (e.g., cloacal malformations, high anorectal malformations with urological involvement) enhances outcomes through shared expertise. Such collaboration can serve as a model for cross-training in institutions with limited subspecialty staff [48].

Institutional examples: In HICs, there are tertiary children's hospitals where both services are available and management of complex cases is based on subspecialty [2]. In LMICs, teaching hospitals may lack pediatric urologists. However, in such instances, pediatric surgeons manage urological cases in collaboration with adult urologists [2].

Challenges to implementation of collaborative models: A key hurdle is institutional resistance or siloed departments, followed by a lack of infrastructure for joint clinics or MDTs. Scheduling and workload alignment, differences in philosophy or approach to certain conditions, and funding and reimbursement issues (in systems with fee-for-service models) also play a role.

Recommendation for practice: To counter the challenges, it is essential to establish formal referral pathways and shared protocols. Joint surgical case reviews and operative planning meetings must be promoted. Regular cross-specialty case conferences should be encouraged. Principles of MDT need to be integrated into training curricula. In resource-limited settings, advocacy should involve visiting urologist programs or tele-collaboration models.

Training, credentialing, and workforce issues

The overlap between pediatric urology and pediatric surgery is significantly shaped by the training pathways, credentialing systems, and workforce realities that differ across regions. These factors influence who performs what procedures, how skills are developed, and how gaps in care are addressed.

Training Pathways

In HICs such as the US and Canada, pediatric urology is a subspecialty of adult urology [18]. Trainees complete a urology residency (five to six years) followed by a one- to two-year pediatric urology fellowship. Pediatric surgery trainees often receive limited exposure to urology unless pursuing further subspecialization [18]. In the UK and Ireland, pediatric surgery is a core surgical specialty with some urology exposure. Pediatric urology can be pursued via fellowships by either pediatric surgeons or urologists [24,49]. The Intercollegiate Board and BAPU influence competency standards. Training pathways vary widely in Europe. Dual-trained specialists (urology and pediatric surgery) exist in some systems. The ESPU aims to standardize training across EU member states [21].

In LMICs, pediatric surgeons often manage urological conditions due to the absence of dedicated pediatric urologists. Formal pediatric urology training is limited or unavailable locally [22,50]. Exposure to complex urology often comes through short-term observerships or international fellowships, visiting subspecialist clinics, and on-the-job learning with limited supervision [51,52].

Credentialing and Scope of Practice

Credentialing bodies often define which procedures are expected competencies for each specialty [53]. For instance, orchidopexy is core for both pediatric surgeons and pediatric urologists. Complex hypospadias repair or PUV ablation may be restricted to pediatric urologists in high-resource settings [45,54]. In many LMICs, the lack of formal credentialing pathways for pediatric urology leads to overlap by necessity. Credentialing challenges include inconsistent operative case requirements during training, no formal maintenance of competence mechanisms in some regions, and ambiguity in surgical audit and outcome tracking by specialty.

Workforce Limitations and Maldistribution

There is a shortage of pediatric urologists in most countries; even in high-income settings, pediatric urologists are concentrated in urban centers [22]. Long waiting times and referral delays are common. Task-shifting to pediatric surgeons occurs frequently to ensure timely care, which may be practical in many LMICs, but it raises concerns about consistency in outcomes, especially for complex procedures [22].

Challenges in Surgical Education and Exposure

There are several challenges to surgical education, including inconsistent exposure to urological procedures during pediatric surgery training [55] and a decline in open urological surgery exposure due to increased use of endoscopy and robotics in high-resource settings [56]. In addition, there is difficulty accessing simulation tools or urodynamics training in many institutions [57]. Furthermore, there are limited mentorship opportunities in pediatric urology in LMICs [51].

*Emerging Solutions* 

Hybrid training models allow dual exposure to pediatric surgery and urology [19]. Meanwhile, fellowships and mentorship programs between high- and low-resource countries can help ease the burden on either specialty. Curriculum reform, integrating more pediatric urology content into pediatric surgical training, is required. Virtual learning should be encouraged via online modules, surgical videos, and live-streamed procedures to enhance exposure [51].

Ethical, logistical, and patient-centered considerations

The interface between pediatric urology and pediatric surgery is not purely clinical or institutional [57]; it has important ethical, logistical, and patient-centered implications. As care models evolve, it is vital to consider how specialty overlaps affect surgical outcomes, family experience, informed consent, and equitable access to care [51].

Ethical Considerations in Specialist Boundaries

Scope of competence and consent: Surgeons must operate within the limits of their training and experience [58]. Ethical dilemmas arise when practitioners manage complex cases (e.g., proximal hypospadias or PUVs) without the appropriate subspecialist expertise. Informed consent should include disclosure of the surgeon’s experience level and available alternatives, including referral to a pediatric urologist when indicated [59]. Parents may not always be aware of the differences between specialists or the potential impact on outcomes [60].

Equity vs. excellence: In low-resource settings, the ethical challenge lies between providing accessible care locally, even if subspecialist input is lacking, and delaying treatment for referral to better-equipped centers [61]. Ethical frameworks must balance justice (equity of access) with non-maleficence [62].

Logistical and System-Level Challenges

Referral pathways: Ambiguous or inefficient referral systems can delay care or result in unnecessary duplication of assessments [63]. Some systems lack clear triage criteria for which patients require specialist review [64,65].

Resource availability: Essential infrastructure (e.g., pediatric endoscopy, urodynamics, specialized nursing) is often limited in non-tertiary centers. Scheduling surgeries between two specialties may be constrained by theater time, staffing, and interdepartmental coordination [66].

Data and outcome tracking: When multiple specialties manage the same conditions, outcome data becomes fragmented [67,68]. This impairs quality assurance, benchmarking, and evidence-based improvement in surgical care.

Patient- and Family-Centered Considerations

Continuity of care: Families value consistency in their child’s care team. Fragmented care (e.g., initial diagnosis by one specialty, surgery by another, and follow-up elsewhere) can create confusion and anxiety [68,69]. A coordinated, multidisciplinary approach fosters trust and improves adherence to postoperative care plans [70,71].

Communication and expectations: Variation in surgical philosophy or technique between pediatric surgeons and urologists (e.g., single-stage vs. two-stage hypospadias repair) can result in mixed messages to families. Standardized counselling protocols and joint clinics can harmonize communication.

Cultural sensitivity and parental preferences: In some communities, families may prefer local care over referral, even if subspecialist options exist elsewhere. Respecting patient autonomy while ensuring safe and appropriate care is a nuanced process, especially when resources are limited.

Strategies to Address Ethical and Patient-Centered Challenges

Role of institutional culture and leadership: Leadership from department heads and institutional boards is critical in encouraging collaboration over competition, building systems that reward teamwork and joint responsibility, and ensuring continuous training in ethical and family-centered care. In conclusion, while the boundary between pediatric urology and pediatric surgery can be clinically flexible, ethical and patient-centered care demands clarity, coordination, and compassion [72].

Recommendations and future directions

As the boundaries between pediatric urology and pediatric surgery continue to evolve, future efforts must focus on creating integrated, adaptable, and patient-centered care models that respond to local realities while adhering to global best practices. Clear frameworks for training, collaboration, and accountability are pivotal to improving outcomes and professional harmony in overlapping domains. Cross-specialty training and exposure should be built into training programs, such as pediatric surgery trainees should receive well-structured rotations in pediatric urology, and vice versa. Simulation-based and case-based learning and discussion should include joint modules on shared procedures (e.g., orchidopexy, hypospadias repair). International curricula harmonization should include the adoption of minimum global training standards in pediatric urology for all surgical trainees managing pediatric genitourinary cases. Regulatory bodies such as the ESPU, BAPU, and ABU should create a cross-certifiable syllabus. Capacity building in LMICs should be geared toward supporting dual-trained surgeons or pediatric surgeons with extended urology competencies. In addition, institutions should invest in international fellowships.

Collaborative Models as the Standard of Care

Joint pediatric urology-surgery clinics and MDTs should be expanded to tertiary centers. Co-management of complex cases should be encouraged, particularly regarding genital reconstruction, neurogenic bladder, and urological malignancies. Shared surgical audits and outcome registries should be promoted to track quality across both specialties.

Clear Referral and Scope-of-Practice Guidelines

Institutions should develop local protocols for when a pediatric surgeon vs. a pediatric urologist should manage a case. Escalation and referral pathways should be based on complexity. National surgical bodies should support scope-of-practice statements that clarify expected competencies and encourage collaboration, not territoriality.

Leveraging Technology and Innovation

A good example is the use of telemedicine for remote consultations between pediatric surgeons and distant pediatric urologists. This would be particularly impactful in rural areas and LMICs. Virtual training platforms, including the use of open-access libraries of operative videos, lectures, and interactive case reviews, should be used to upskill generalists. Robotic and endoscopic skills training would help promote hands-on workshops and international collaboration, thus expanding access to modern pediatric urology tools.

Advocacy and Health Policy

Advocacy would help to expand the workforce in pediatric urology where needed. Policymakers should be reminded of the importance of funding subspecialty training, the use of multidisciplinary service models, ensuring equitable pediatric surgical access, and including pediatric urology and surgery interface discussions in national and regional surgical planning strategies.

Research and Data-Driven Improvements

Improvements can be made using research-proven findings, such as outcomes of shared procedures in both specialties, such as hypospadias repair or orchidopexy. Also, comparative studies of models of care, such as urology-led or surgery-led models, develop regional and global databases for pediatric urology outcomes. Research activities could focus on long-term follow-up, patient outcomes, and the effectiveness of collaborative models. 

## Conclusions

The relationship between pediatric urology and pediatric surgery is a dynamic and evolving area within pediatric surgical care. Though these specialties differ in their training and focus, they often overlap in treating various congenital and acquired genitourinary conditions in children. This intersection is especially evident in procedures like hypospadias repair, orchidopexy, and the treatment of PUVs, conditions that may be handled by either specialty depending on institutional resources, training, and regional workforce availability. While this overlap can sometimes create ambiguity, it also fosters opportunities for collaboration, innovation, and comprehensive patient care.

As healthcare systems strive to provide more effective, efficient, and equitable care by integrating multidisciplinary approaches such as joint clinics, co-managed surgeries, and shared training programs, it becomes both a practical and ethical necessity. Standardizing training, clarifying credentialing, and addressing workforce shortages, particularly in LMICs, are crucial steps to minimizing outcome variability and ensuring children receive timely, expert care. Ultimately, the aim is not to enforce rigid divisions but to promote mutual respect, teamwork, and shared accountability between pediatric surgeons and urologists. By strategically investing in training, infrastructure, and policy, the boundary between these specialties can shift from a potential conflict to a foundation for partnership, enhancing care for children in diverse clinical and geographic contexts.

## References

[1] O’Kelly O’Kelly, D. F. (2019, October 10 (2019). Paediatric urology: a subspecialty born from collaboration. https://paediatricurology.ie/paediatric-urology-a-subspecialty-born-from-collaboration/.

[2] deVries CR (2022). A global view of pediatric urology. J Pediatr Urol.

[3] Losty PD (1999). Recent advances: paediatric surgery. BMJ.

[4] Reynard J, Mark S, Turner K (2011). Paediatric urology. Urological Surgery.

[5] Santos JD, Farhat WA, Shouldice M (2025). The integral co-management role of the medical pediatric urologist: improving the care of children with urological conditions. J Pediatr Urol.

[6] Danzig MR, Alpert SA, Copp HL (2024). The development of surgical ability during pediatric urology fellowship and its evolution in the early years of practice. J Pediatr Urol.

[7] Wang MH, Chen B, Kern D, Gearhart S (2013). Pediatric urology fellowship training: are we teaching what they need to learn?. J Pediatr Urol.

[8] Kenny SE (2021). Paediatric General Surgery and Urology. https://gettingitrightfirsttime.co.uk/wp-content/uploads/2021/09/PaediatricSurgeryReport-Sept21w.pdf.

[9] Brock C (2023). The child surgical patient in the early twentieth century. J Hist Med Allied Sci.

[10] Cunningham H (2005). Children and Childhood in Western Society Since 1500.

[11] Losty PD (2025). Paediatric surgery. BMJ.

[12] Spitz L (2012). The history of paediatric surgery in the United Kingdom and the influence of the national health service on its development. J Pediatr Surg.

[13] (2025). History of Pediatric Surgery | PedSurg Resource. https://www.pedsurglibrary.com/apsa/view/PedSurgResource/1884037/all/History_of_Pediatric_Surgery?refer=true.

[14] Rushton HG, Kaplan GW, Mitchell ME, Caldamone AA (2019). Road to pediatric urology subcertification: a 25-year journey. J Pediatr Urol.

[15] Innes Williams D (2003). The history of paediatric urology: personal recollections 1948-1978. BJU Int.

[16] Koyle MA (2017). Pediatric urology: then and now. Can Urol Assoc J.

[17] Radmayr C, Tekgul S (2017). Paediatric Urology and the dilemma of low-quality evidence for the management of common urological conditions (vesicoureteral reflux, lower urinary tract dysfunction, undescended testis) in children. Eur Urol Focus.

[18] (2025). Residency Requirements | The American Board of Urology. https://abu.org/residency-requirements/.

[19] (2025). So you want to be a paediatric surgeon? | British Association of Paediatric Surgeons. https://www.baps.org.uk/trainees/prospective-trainees/want-to-be-paediatric-surgeon/.

[20] (2025). The British Association of Paediatric Urologists—Home. https://www.bapu.org.uk/.

[21] (2025). About ESPU - ESPU | European Society for Paediatric Urology. https://www.espu.org/about-espu/.

[22] (2022 ). Paediatric urology in Sub‐Saharan Africa: challenges and opportunities. BJU Int.

[23] Groth TW, See WA, Ramsay S, Cooper CS, Kryger JV (2018). The implications of fellowship expansion on future pediatric urologist surgical volumes. J Pediatr Urol.

[24] Robb Robb, M. A. (2019, February 19 (2019). What is paediatric urology?. https://www.paedsurology.com/what-is-paediatric-urology/.

[25] Van Batavia JP, Shukla AR, Joshi RS, Reddy PP (2018). Pediatric urology and global health: why now and how to build a successful global outreach program. Urol Clin North Am.

[26] Donaire AE, Mendez MD (2025). Hypospadias. http://www.ncbi.nlm.nih.gov/books/NBK482122/.

[27] Halaseh SA, Halaseh S, Ashour M (2022). Hypospadias: a comprehensive review including its embryology, etiology and surgical techniques. Cureus.

[28] Wu Y, Guan Y, Wang X, Wang C, Ma X, Guan H (2023). Repair of proximal hypospadias with single-stage (Duckett's method) or Bracka two-stage: a retrospective comparative cohort study. Transl Pediatr.

[29] Bhandarkar Bhandarkar, K. K., & Garriboli, M. (2019 (2019). Repair of distal hypospadias: cosmetic or reconstructive?. EMJ Urol.

[30] Bickler SW, Kyambi J, Rode H (2001). Pediatric surgery in sub-Saharan Africa. Pediatr Surg Int.

[31] Derbew M (2019). Pediatric surgery in Eastern Africa: the unmet need. J Pediatr Surg.

[32] Özman O, Kuru M, Gezer M (2019). Outcomes of hypospadias surgery performed by different surgeons under the supervision of an experienced pediatric urology surgeon. J Urol Surg.

[33] Fenner A (2010). Pediatrics: 20 years of hypospadias repair—yet still no consensus. Nat Rev Urol.

[34] Vallasciani S, Minoli DG, Manzoni G (2015). Hypospadias repair: the ongoing challenge. Pediatric Urology: Contemporary Strategies from Fetal Life to Adolescence.

[35] Hsu HS (2012). Management of boys with nonpalpable undescended testes. Urological Science.

[36] Moursy EE, Gamal W, Hussein MM (2011). Laparoscopic orchiopexy for non-palpable testes: outcome of two techniques. J Pediatr Urol.

[37] Pettersson A, Richiardi L, Nordenskjold A, Kaijser M, Akre O (2007). Age at surgery for undescended testis and risk of testicular cancer. N Engl J Med.

[38] Strader CH, Weiss NS, Daling JR, Karagas MR, McKnight B (1988). Cryptorchism, orchiopexy, and the risk of testicular cancer. Am J Epidemiol.

[39] Herrinton LJ, Zhao W, Husson G (2003). Management of cryptorchism and risk of testicular cancer. Am J Epidemiol.

[40] Yousef Y, Lee A, Ayele F, Poenaru D (2019). Delayed access to care and unmet burden of pediatric surgical disease in resource-constrained African countries. J Pediatr Surg.

[41] Chan YY, Durbin-Johnson B, Kurzrock EA (2016). Pediatric inguinal and scrotal surgery — practice patterns in U.S. academic centers. J Pediatr Surg.

[42] Coquillette M, Lee RS, Pagni SE, Cataltepe S, Stein DR (2020). Renal outcomes of neonates with early presentation of posterior urethral valves: a 10-year single center experience. J Perinatol.

[43] Brownlee E, Wragg R, Robb A, Chandran H, Knight M, McCarthy L (2019). Current epidemiology and antenatal presentation of posterior urethral valves: outcome of BAPS CASS National Audit. J Pediatr Surg.

[44] Thakkar D, Deshpande AV, Kennedy SE (2014). Epidemiology and demography of recently diagnosed cases of posterior urethral valves. Pediatr Res.

[45] Bingham G, Leslie SW, Rentea RM (2025). Posterior Urethral Valves. http://www.ncbi.nlm.nih.gov/books/NBK560881/.

[46] Talabi AO, Sowande OA, Etonyeaku AC, Salako AA, Adejuyigbe O (2015). Posterior urethral valves in children: pattern of presentation and outcome of initial treatment in Ile-Ife, Nigeria. Niger J Surg.

[47] Browne C, Dowling CM, O'Malley P (2018). Outcomes from the introduction of a combined urology outpatient clinic. Adv Urol.

[48] Levitt MA, Peña A (2010). Cloacal malformations: lessons learned from 490 cases. Semin Pediatr Surg.

[49] (2025). Paediatric urology sub-specialty fellowship | Health Education Yorkshire and Humber. https://www.yorksandhumberdeanery.nhs.uk/recruitment/medical-recruitment-school-surgery/paediatric-urology-sub-specialty-fellowship.

[50] Trail Trail, M. M., & Aslam, M. Z. (2024 (2024). Urological surgery training in low- and low-middle-resource settings: a model for success!. Curr Bladder Dysfunct Rep.

[51] Baqain L, Haddad S, Baqain R (2024). Post-graduate urology training in low- and middle-income countries. Soc Int Urol J.

[52] Metzler I, Bayne D, Chang H, Jalloh M, Sharlip I (2020). Challenges facing the urologist in low- and middle-income countries. World J Urol.

[53] McElnay PJ, McGoldrick C, Beamish AJ, Hoo C, Gokani VJ, Harries RL (2016). Credentialing in surgical specialities: recommendations by the Association of Surgeons in Training. Int J Surg.

[54] Woodhouse CR, Neild GH, Yu RN, Bauer S (2012). Adult care of children from pediatric urology. J Urol.

[55] Cleaveland P, Jones C, Thompson A (2010). Exposure to paediatric urology during urology specialty training: a UK national trainee survey. Bull R Coll Surg Engl.

[56] Watson G, Niang L, Chandresekhar S, Natchagande G, Payne SR (2022). The feasibility of endourological surgery in low-resource settings. BJU Int.

[57] Taghavi K (2025). What's new in paediatric urology? Crossroads of pediatric surgery and adult urology. Ther Adv Urol.

[58] sitecore\ddean@rcseng.ac.uk. (n.d.). Clinical Governance of the Surgical Care Team (2025). Clinical governance of the surgical care team - Royal College of Surgeons of England. https://www.rcseng.ac.uk/standards-and-research/standards-and-guidance/service-standards/surgical-care-team-guidance/clinical-governance/.

[59] Burger I, Schill K, Goodman S (2007). Disclosure of individual surgeon's performance rates during informed consent: ethical and epistemological considerations. Ann Surg.

[60] Faden RR, Beauchamp TL, King NMP (1986). A History and Theory of Informed Consent. https://bioethics.jhu.edu/wp-content/uploads/2022/05/Beauchamp-TL-Faden-RR-Meaning-and-Elements-of-Informed-Consent.pdf.

[61] Provenzano AM, Khoshnood K (2010). Caring for patients in low-resource settings. Virtual Mentor.

[62] Varkey B (2021). Principles of clinical ethics and their application to practice. Med Princ Pract.

[63] Rathnayake D, Clarke M (2021). The effectiveness of different patient referral systems to shorten waiting times for elective surgeries: systematic review. BMC Health Serv Res.

[64] Rai S, Kelly MJ (2007). Prioritization of colorectal referrals: a review of the 2-week wait referral system. Colorectal Dis.

[65] Cerdán Carbonero MT, Sanz López R, Martínez Ramos C (2005). Improving communication between levels of health care: direct referral of patients to a one-stop service for major outpatient surgery. Aten Primaria.

[66] May JH, Spangler WE, Strum DP (2011). The surgical scheduling problem: current research and future opportunities. Prod Oper Manag.

[67] Kailasam M, Guo W, Hsann YM, Yang KS (2019). Prevalence of care fragmentation among outpatients attending specialist clinics in a regional hospital in Singapore: a cross-sectional study. BMJ Open.

[68] Haggerty JL, Freeman GK, Adair CE (2025). Continuity of care: a multidisciplinary review. BMJ.

[69] Epstein K, Juarez E, Epstein A, Loya K, Singer A (2010). The impact of fragmentation of hospitalist care on length of stay. J Hosp Med.

[70] Taberna M, Gil Moncayo F, Jané-Salas E (2020). The multidisciplinary team (MDT) approach and quality of care. Front Oncol.

[71] Bendowska A, Baum E (2023). The significance of cooperation in interdisciplinary health care teams as perceived by Polish medical students. Int J Environ Res Public Health.

[72] Bosch B, Mansell H (2015). Interprofessional collaboration in health care: lessons to be learned from competitive sports. Can Pharm J.

